# A Cohort Study on Alzheimer's Disease in Relation to Residential Magnetic Fields From Indoor Transformer Stations

**DOI:** 10.1002/bem.70031

**Published:** 2025-10-31

**Authors:** Anu Liimatainen, Päivi Roivainen, Jukka Juutilainen, Anne Höytö, Jonne Naarala

**Affiliations:** ^1^ Department of Environmental and Biological Sciences University of Eastern Finland Kuopio Pohjois‐Savo Finland; ^2^ Radiation Practices Regulation Radiation and Nuclear Safety Authority Vantaa Uusimaa Finland

**Keywords:** Alzheimer's disease, electromagnetic fields, environmental exposure, epidemiology, nonionizing radiation

## Abstract

Meta‐analyses of epidemiological studies have suggested that Alzheimer's disease (AD) may be linked with exposure to extremely low frequency (ELF) magnetic fields (MF). This is the first study investigating the association of AD with exposure to residential ELF MFs from indoor transformer stations, using a study design that avoids shortcomings of previous studies. All cohort members had lived in buildings with indoor transformer stations. MF exposure was assessed based on the location of their apartment in relation to the transformer room. AD patients were identified from Drug Purchase Register and Drug Reimbursement Register. Out of the 155,562 individuals, 5652 (111,357 person‐years of follow‐up) living in apartments next to transformer stations were considered as exposed, while 115,772 (2,289,526 person‐years of follow‐up) individuals living on higher floors of the same buildings were considered as referents. Associations between MF exposure and AD were examined using Cox proportional hazard models. The hazard ratio (HR) was 1.02 (95% confidence interval: 0.85–1.22), indicating that the risk of AD is not associated with residential ELF MFs present in apartments next to transformer stations. The duration of residence did not essentially change the HR. The risk of AD was slightly but not statistically significantly higher (HR 1.22, 95% confidence interval: 0.94–1.57) for those whose residence started before the age of 50 years. The results did not support positive findings from previous studies that have reported a link between AD and occupational or residential MF exposure. Bioelectromagnetics. 00:00–00, 2025.

## Summary

- The study included high residential extremely low frequency (ELF) magnetic field (MF) levels from indoor transformer stations and avoided many limitations of previous studies.
- Residential ELF MFs did not affect the risk of Alzheimer's disease (AD).
- Further studies should focus on investigating occupational ELF MF exposure and the potential effect of diurnal timing of MF on the risk of AD.

## 1 Introduction

Human exposure to environmental extremely low frequency (ELF) magnetic fields (MF) occurs wherever electricity is generated, transmitted, distributed or used. Several epidemiological studies have investigated health risks in populations living near power lines, in electrical occupations and in other groups exposed to ELF MFs. ELF MFs have been classified as possibly carcinogenic to humans mainly based on findings suggesting increased risk of childhood leukemia (IARC 2002).

Concerning other diseases than cancer, the evidence is strongest, although inadequate and inconsistent, for an association between Alzheimer's disease (AD) and ELF MFs (WHO World Health Organization 2007), with most of the existing studies focusing on occupational exposure. Meta‐analyses have provided some support for an association between occupational MF exposure and AD (Garcia et al. 2008; Jalilian et al. 2018; Vergara et al. 2013). However, many methodological issues (e.g. exposure assessment, verifying diagnoses) may have affected the results of independent studies (Röösli 2008). Three studies have addressed residential exposure using distance to power lines as an exposure metric. Huss et al. (2009) found that the risk of AD among people living within 50 m from power lines increased with duration of residence next to power lines. Gervasi et al. (2019) reported an odds ratio of 1.11 (95% CI 0.95–1.30) for individuals living at distances < 50 m from power lines, while Frei et al. (2013) observed no overall increase in the risk of AD in persons living within 50 m from power lines although there was a weak suggestion of an increased risk when AD was diagnosed by the age of 75 years. Exposure assessment is a problem in these studies, as distance to power lines is only a crude estimate of MF exposure.

The aim of the present study was to investigate whether residential exposure to ELF MFs increases the risk of AD. We conducted a cohort study using a unique database of residential buildings containing indoor transformer stations. This database provides an opportunity to explore potential health effects of ELF MFs with an advanced study approach featuring significant exposure levels, avoidance of selection bias, and minimized potential for confounding factors (Khan et al. 2020). This database has previously proved to be useful in showing associations of residential ELF MFs with hematological malignancies (Crespi et al. 2024; Khan et al. 2021), brain tumors (Khan et al. 2021), skin cancers (Khan et al. 2022) and digestive organ cancers (Juutilainen et al. 2024).

## 2 Materials and Methods

### 2.1 Participants

The study cohort was identified using the Database of Finnish Buildings with Indoor Transformer Stations (DaFBITS). This database, compiled by our research group, was described in detail in Khan et al. (2020). It includes information on buildings with indoor transformer stations from electricity distribution companies and building control offices. Data on individuals who lived in these buildings, including dates of moving in or out as well possible dates of death and emigration was collected from the Population Information System, maintained by the Finnish Digital and Population Data Services Agency. The computer‐based Population Information System began in 1971 and has extensive residential records from 1983 onward, with limited earlier data. Follow‐up for residence in the buildings started on the earliest available date for each individual. The University of Eastern Finland's Ethical Committee approved the study protocol in January 2017 (Statement 4/2017). No informed consents were needed as the study used only register data without direct contact with the subjects, in accordance with Finnish regulations.

### 2.2 Exposure Assessment

Exposure assessment was based on data stored in DaFBITS. Apartments were classified into five ELF MF exposure categories (Khan et al. 2020) based on their proximity to the transformer room, which is always on the ground floor or in the basement. These transformers convert 50 Hz voltage from 10 kV or 20 kV to 230 V, serving multiple buildings. An individual was classified as “exposed” if she/he lived for at least 6 months in an apartment directly above (category 1) or sharing a wall with the transformer room (category 2). Persons living in apartments sharing only a corner with the transformer room (category 3) were excluded due to the small group size and lack of exposure measurements. The reference group included individuals who lived for at least 6 months in apartments on any floor other than the first or ground floors (category 5). We also compared the residents of other first or ground‐floor apartments (category 4) with referents to assess possible confounding related to living on the lowest floors. The ELF MF exposure level measured in 30 Finnish buildings was on the average 0.56 µT (variation from 0.17 to 1.55 µT) in category 1 apartments, 0.21 µT (0.03–0.62) in category 4 apartments and 0.10 µT (0.02–0.70) in category 5 apartments (Ilonen et al. 2008).

### 2.3 Outcome Assessment

The cohort was linked to registers maintained by Social Insurance Institution of Finland (Kela) using unique personal identifiers assigned to each Finnish resident to obtain information on AD diagnoses. Drug Reimbursement Register can be used to find AD patients with Finnish AD drug reimbursement code 307. Using international Anatomical Therapeutic Chemical (ATC) ‐codes for AD drugs we also collected information from Drug Purchase Register. We used ATC‐codes N06DA01 tacrine, N06DA02 donepezil, N06DA03 rivastigmine, N06DA04 galantamine, combined code N06DA52 donepezil and memantine, and N06DX01 memantine to identify AD patients. These two registers complement each other and make identifying of AD patients more reliable. AD registers started from January 1, 1999, which is the earliest date for AD diagnosis in this study.

### 2.4 Follow‐Up

All study participants were aged 30 or older when follow‐up started and had resided in buildings included in DaFBITS. Follow‐up began 6 months after moving into the apartment and continued until December 31, 2016, emigration, death, or AD disease diagnosis. For individuals younger than 30 at the start of residence, follow‐up began at the age of 30 years (after 6 months of residence). If the move‐in date was before January 1, 1971 (the year Finland's computer‐based Population Information System was introduced) the follow‐up began 6 months later (July 1, 1971). If the transformer was installed after the resident had already moved in, the follow‐up started 6 months after the installation date.

If a person living in a reference category apartment later moved to an apartment of higher exposure, her/his exposure status was reassessed. Thus, a person could have had different exposure statuses during the stay in the study buildings. However, the status did not change as a result of moving from a more exposed to a less exposed apartment, as living once in an exposed apartment (for at least 6 months) sufficed for classification as an exposed person.

### 2.5 Characteristics of the Participants

The cohort included 155,562 individuals, of whom 73,725 (47.4%) were men and 81,837 (52.6%) women. Their median age at the start of residence was 30.0 years with interquartile range (IQR) from 24.0 to 41.0 years. Of these, 5652 (3.6%) were in the exposed group, and 115,772 (74.4%) lived in reference apartments (Table 1). Median duration of residence varied from 3.9 to 4.4 years in the apartment categories included in the analyses (Table 1). The total person‐years of follow‐up were 111 357 for the exposed residents, 2,289,526 for the referents and 698,991 for the first or ground floor residents.

**Table 1:** Extremely low frequency magnetic field exposure categories (Khan et al. 2020) and characteristics of the study individuals in these categories. Total *N* = 155,562. Number (and percentage) is shown for sex and median (and 5th to 95th percentile) for other variables.

Apartment category	1	2	4	5
Number of individuals	5234	418	34,138	115,772
Male: *N* (%)	2527 (48.3)	211 (50.5)	16,311 (47.8)	54,676 (47.2)
Female: *N* (%)	2707 (51.7)	207 (49.5)	17,827 (52.2)	61,096 (52.8)
Age at the start of residence (years)	31 (6–61)	31 (11–61)	30 (8–62)	30 (7–61)
Duration of residence (years)	4.0 (0.7–24.7)	4.3 (0.9–27.3)	3.9 (0.7–24.8)	4.4 (0.7–29.6)

*Note:* 1 = apartment located above the transformer room (exposed); 2 = apartment sharing a wall with the transformer room (exposed); 4 = apartment located on the same floor as apartment in category 1 or 2 (first or ground floor residents); 5 = apartment located on any other floor of the building (referents). Category 3 was excluded because of small group size and lack of exposure measurements.

The median person‐years of follow‐up was 18.8 years (IQR from 11.8 to 26.9) for the exposed group and 18.7 years (IQR from 11.7 to 27.2) for the reference group. For the first or ground floor residents the median person‐years of follow‐up was 18.2 years (IQR from 11.7 to 26.1).

The total number of AD patients living in these apartments was 3663, of whom 1341 (36.6%) were men and 2322 (63.4%) women. Their median age at the start of the residence was 51.5 years (IQR from 42.0 to 62.0). Of these, 123 (3.4%) were in the exposed group, and 2810 (76.7%) lived in the reference apartments.

### 2.6 Statistics

Analyses were planned beforehand. Cox proportional hazard models were used to estimate hazard ratios (HR) with 95% confidence intervals (CI) for the association of residential exposure with AD, using time on study (in years) as the time scale. Results were adjusted for sex and age at the start of residence. Analyses were conducted using IBM SPSS Statistics Version 29 (IBM Corp, Armonk NY, USA). Statistical significance of the difference between two HR values was tested as described by Altman and Bland (2003).

To investigate possible dependence of AD risk on the duration of MF exposure, Cox models were applied to individuals who had lived in the study residences for ≥ 3 years or ≥ 10 years, starting follow‐up after the specified minimum duration of 6 months. To study the effect of age at the onset of exposure, Cox models were run separately for individuals who had started living in residences when they were either less than 50 years or at least 50 years old.

As a sensitivity analysis category 2 apartments (sharing a wall with the transformer room) were excluded from the Cox model. Although no exposure measurements in Finland are available for these apartments, they were included in the main analysis, as measurements performed in other countries indicate that levels of ELF MFs are elevated (although lower than those in category 1 apartments) in category 2 apartments (Huss et al. 2013; Röösli et al. 2011). To assess potential confounding from living on the first or ground floor, risk of AD was estimated also for individuals who lived for at least 6 months in apartments on these floors but not adjacent to the transformer room (category 4), referred to as “first or ground floor residents.”

## 3 Results

### 3.1 Exposed Residents

The HR for AD was close to 1.00, indicating that the risk of AD is not associated with residential ELF MFs present in apartments next to transformer stations (Table 2). The HR was essentially unchanged in the sensitivity analysis excluding category 2 apartments. Restricting the analysis to those who had lived in the apartments for at least 3 years or at least 10 years resulted in only slightly higher HRs.

**Table 2:** Hazard ratios (HR) and 95% confidence intervals (95% CI) for Alzheimer's disease among persons exposed to extremely low frequency magnetic fields from indoor transformer stations. Adjusted for sex and age. The total person‐years of follow‐up were 111,357 for exposed and 2289 526 for referents.

	Exposed cases	Referent cases	HR	95% CI
Main analysis	123	2810	1.02	0.85–1.22
Sensitivity analysis^a^	117	2810	1.01	0.84–1.22
Duration of exposure ≥ 3 years	104	2313	1.07	0.88–1.30
Duration of exposure ≥ 10 years	66	1519	1.10	0.85–1.41
Age at the start of residence < 50 years	62	1282	1.22	0.94–1.57
Age at the start of residence ≥ 50 years	61	1528	0.91	0.70–1.17

a Category 2 apartments excluded.

### 3.2 First or Ground Floor Residents

The HRs calculated for the residents of lowest floors showed no clear evidence of such confounding that would affect (increase or decrease) the AD risk of first or ground floor residents (and thereby also the risk of the MF‐exposed subjects) (Table 3). It may be of interest that the HR increased slightly in analyses restricted to those who had lived in the apartments for at least 3 or at least 10 years, as a similar pattern was observed among the MF‐exposed individuals.

**Table 3:** Hazard ratios (HR) and 95% confidence intervals (95% CI) for Alzheimer's disease among residents of such first or ground floor (FGF) apartments that were not located next to transformer stations (category 4 apartments). Adjusted for sex and age. The total person‐years of follow‐up were 698,991 for FGF and 2,289,526 for referents.

	FGF cases	Referent cases	HR	95% CI
All study subjects included	730	2810	1.05	0.97–1.14
Duration of exposure ≥ 3 years	581	2313	1.06	0.97–1.16
Duration of exposure ≥ 10 years	341	1519	1.07	0.95–1.21
Age at the start of residence < 50 years	305	1282	1.02	0.90–1.15
Age at the start of residence ≥ 50 years	425	1528	1.08	0.97–1.20

### 3.3 Age at the Start of Residence

Age at the start of residence in an exposed apartment affected the risk estimate only slightly. The HR was 1.22 (95% CI 0.94–1.57) for those whose residence started before the age of 50 years, and 0.91 (95% CI 0.70–1.17) for those who were older than 50 years at the start of residence (Table 2). The difference between these two HRs is not statistically significant (*p* = 0.11). The HRs calculated for the residents of the lowest floors showed no evidence of such confounding that would affect the risk of AD among the subjects whose residence started before the age of 50 years (Table 3).

## 4 Discussion

This was the first study investigating the association of AD with exposure to residential ELF MFs from indoor transformer stations. The results did not show increased risk of AD among persons exposed to ELF MFs, failing to support positive findings from previous studies that have addressed occupational MF exposure or residential MF exposure from power lines.

This study avoided many shortcomings of previous studies. The strengths of the present study included basic design of the study (cohort study), large study size, high exposure levels, validated exposure assessment method (Huss et al. 2013; Ilonen et al. 2008; Röösli et al. 2011), elimination of selection bias (as no contact with the study participants was required) and small risk of confounding (as the exposed persons and referents lived in the same buildings).

Confounding factors can either increase or decrease the observed risk estimate, depending on whether the confounding factor decreases or increases the risk of the disease being studied. As the HRs observed in this study were close to 1.0, we need to be particularly concerned about such confounders that could decrease the risk estimate and thereby obscure a true risk. As pointed out above, the risk of confounding is small because of the study design. Some residual confounding might be related to the fact that all “exposed” individuals lived on the lowest floors of the buildings. However, the most obvious differences between the lower and higher floors (e.g., higher socioeconomic status on higher floors due to slightly higher apartment prices, or higher air pollution levels on lower floors) would increase rather than decrease the risk of AD (Zhang et al. 2021). Importantly, we were able to address any confounding by investigating the risk of AD among such first or ground floor residents who did not live next to a transformer station. No evidence of confounding was found.

As the residences were not visited during the study, we do not have any measurements of possible confounding factors specific to living next to a transformer station, such as noise or vibration. However, no distinguishable noise or vibration was observed in earlier MF measurements in apartments above transformer stations (Ilonen et al. 2008). Moreover, it seems unlikely that weak noise or vibration would bias the risk estimates towards null.

The limitations of the study included lack of information about exposure to ELF MFs from sources other than transformer stations. Measurements performed in buildings with transformer stations (Ilonen et al. 2008) indicate that other residential sources are not likely to be important in comparison to transformer stations, but some members of the cohort may have been exposed to relatively high MFs in occupational settings. Furthermore, information about possible personal confounding factors (e.g., genetic and dietary factors) was not available. Personal confounders or occupational MF exposure could potentially bias the risk estimate, if they were associated with the exposure categories used. For example, if occupational MF exposure were considerably more common in the referent group (category 5) than among the “exposed” subjects, this could decrease the observed risk estimate. However, there is no reason to assume that living next to an indoor transformer station would be associated with personal confounding factors or occupational ELF MF exposure.

The outcome assessment of this study was based on the Drug Reimbursement Register and Drug Purchase Register. These registers complement each other. The use of information on AD drug use in identifying AD patients is more reliable than the commonly used cause‐of‐death registers (Huss et al. 2009; Röösli et al. 2007). Drug Reimbursement Register has good accuracy in showing the presence or absence of AD: a sensitivity of 63.5% and a specificity of 99.9% was reported for the time interval 1999‐2008 (Solomon et al. 2014). While unbiased risk estimates can be obtained when misclassification of outcome is non‐differential and specificity is 100%, even small deviations from perfect specificity can bias the risk estimate towards the null (no effect) (Ma et al. 2024). However, the simulations by Ma et al. (2024) using a specificity of 99.8%, indicate that misclassification of outcome cannot bias any meaningful effect size so much that bias would explain the (nearly) null risk estimates observed in the present study. Imperfect sensitivity (63.5% in this study) introduces little or no bias to the risk estimates, when sensitivity does not markedly differ between the exposed and unexposed groups. Some differential sensitivity of outcome assessment between the exposed and unexposed groups may arise by chance alone, but large cohort size (like that used in the present study) reduces random differences in sensitivity and therefore attenuates the impact of such errors on the risk estimate (Ma et al. 2024).

Also, misclassification of exposure can bias risk estimates towards the null. As shown by Ilonen et al. (2008) exposure assessment based on the location of transformer stations results in low level of exposure misclassification (high specificity in particular). This indicates that marked bias towards the null is not likely in the present study.

There was a slight increasing trend of the HR with increasing duration of exposure (from 1.02 with exposure durations ≥ 6 months to 1.10 with exposure durations of ≥ 10 years). However, the 95% CIs overlapped widely, suggesting that the seeming trend is related to random variation rather than an exposure‐response relationship. Furthermore, a similar trend, albeit weaker, was seen among the first or ground floor residents.

There is no solid information on biologically relevant MF exposure duration. Six months was used as the minimum duration of residence to be included in the study. This selection was motivated by our previous findings from studies focusing on cancer. We used 1 month exposure threshold in the first study on hematological cancers and brain tumors in our group (Khan et al. 2021), but 6 months in later studies (skin cancers, Khan et al. 2022; all adult cancers, Juutilainen et al. 2024), because alternative analysis (unpublished) of the first study results suggested stronger association with ≥ 6 month exposure than with ≥ 1 month exposure.

Previous studies addressing the association between AD and residential ELF MFs have used distance from power lines as the proxy for MF exposure (Frei et al. 2013; Gervasi et al. 2019; Huss et al. 2009). As the magnetic flux densities in apartments next to indoor transformer stations are considerably stronger than those in residence near power lines, our results indicate that the previous positive findings may be explained by confounding and/or other methodological shortcomings rather than a causal link between AD and exposure to residential ELF MF. Comparison of our results to studies based on occupational MF exposure is less straightforward. Magnetic flux densities exceeding those found in apartments above transformer stations do exist in many workplaces. However, many studies on occupational exposure have reported increased risks of AD using cut‐off points of 0.2–0.5 µT for measured or estimated MF level (Davanipour et al. 2007; Feychting et al. 1998, 2003; Håkansson et al. 2003; Sobel et al. 1995, 1996) which is not high in comparison to the mean time‐average exposure of 0.56 µT found in apartments above transformer stations (Ilonen et al. 2008; Khan et al. 2020). Furthermore, according to the review and meta‐analysis by Jalilian et al. (2018), negative findings have been reported in some studies with higher cut‐offs, and the risk estimate does not appear to increase with increasing cut‐off point between 0.2 and 0.5 µT. Due to the imprecision of exposure assessment in occupational exposure studies (Jalilian et al. 2018; Vergara et al. 2013) it is, of course, possible that many members of the “exposed” occupational groups have experienced MF levels that are considerably higher than the cut‐off points used in the epidemiological studies. It is worth noting that very high MF exposures (e.g., mean annual exposure of about 21 µT among train drivers) were reported in a study that found increased risk of AD mortality among railway employees (Röösli et al. 2007). Apart from possible differences in exposure level, another difference between residential and occupational exposure is the diurnal timing of exposure: occupational exposure occurs mainly during the day, while residential exposure of the working‐age population occurs mainly in the evening and at night. There is evidence that MF exposure can influence circadian rhythms (Juutilainen et al. 2018), and disruption of the circadian timing system seems to be linked with the development of AD (Hoyt and Obrietan 2002). However, only few studies have investigated whether MF effects on humans or animals depend on the diurnal timing of MF exposure (Choi et al. 2003; Schuz et al. 2007). There is some evidence that day‐time occupational MF exposure affects nocturnal excretion of melatonin (Juutilainen et al. 2000), possibly interacting with night‐time exposure to light (Juutilainen and Kumlin 2006). Studies with experimental animals have failed to provide support for adverse effects on the development of AD by ELF electromagnetic fields (Hu et al. 2016; Liebl et al. 2015; Liu et al. 2015; Zhang et al. 2013; Zhang et al. 2015; Zuo et al. 2018).

## 5 Conclusions

No evidence for an association between residential ELF MFs and AD was found in this study that involved relatively high exposure levels and avoided many limitations of previous studies addressing residential MF exposure. We recommend that, if any further studies on the possible link between ELF MF exposure and AD are conducted, they should focus on occupational MF exposure, trying to identify occupational groups exposed to particularly strong ELF MFs. Studies (including experimental studies) on the possible differences between night‐time and day‐time exposure might also be helpful.

## Ethics Statement

The University of Eastern Finland's Ethical Committee approved the study protocol in January 2017 (Statement 4/2017). No informed consents were needed as the study used only register data without direct contact with the subjects, in accordance with Finnish regulations.

## Consent

The authors have nothing to report.

## Conflicts of Interest

The authors declare no conflicts of interest.

## Data Availability

Data may be obtained from a third party and are not publicly available. The authors are not permitted to share the data collected from companies and Finnish national registers. The data can be obtained directly from these parties according to their regulations.
